# Atypical Kartagener syndrome in a 40-year-old woman: Computed Tomography findings of situs inversus with bronchiectasis and infertility

**DOI:** 10.1016/j.radcr.2026.06.087

**Published:** 2026-07-09

**Authors:** Yohannes Girma Zewdie, Biniyam Beyene Tabor, Ermias Fikru Yesuf, Hana Yeshewas Genetirune

**Affiliations:** a Department of Radiology, Addis Ababa University, Addis Ababa, Ethiopia; b Department of Radiology, Saint Paul’s Hospital Millennium Medical College, Addis Ababa, Ethiopia; c Department of Surgery, Debre Berhan University, Debre Berhan, Ethiopia; d Department of Anesthesia, Jimma University, Jimma, Ethiopia

**Keywords:** Primary ciliary dyskinesia, Kartagener syndrome (atypical), Situs inversus, Bronchiectasis, Infertility, Computed tomography

## Abstract

Primary ciliary dyskinesia is a rare inherited disorder characterized by impaired mucociliary clearance leading to recurrent respiratory infections. Kartagener syndrome represents a subset of this condition classically defined by the triad of bronchiectasis, chronic sinusitis, and situs inversus, although atypical presentations lacking one component of the triad may occur. We report the case of a 40-year-old female who presented with productive cough, shortness of breath, chest pain, and fever, with a long-standing history of recurrent respiratory infections since adolescence that showed only temporary response to antibiotic therapy. The patient also reported primary infertility. Computed tomography of the chest demonstrated dextrocardia with complete situs inversus, bilateral bronchiectasis predominantly involving the middle and lingular lobes, mucus impaction, and associated tree-in-bud nodularity with ground-glass opacities. There was no clinical or radiologic evidence of sinusitis. These findings are consistent with an atypical presentation of Kartagener syndrome. This case highlights the importance of recognizing characteristic imaging features and considering underlying ciliary disorders in patients with recurrent respiratory infections and situs anomalies, even in the absence of sinus disease.

## Introduction

Primary ciliary dyskinesia is a genetically heterogeneous disorder resulting from structural and functional abnormalities of motile cilia, leading to impaired mucociliary clearance and chronic or recurrent respiratory tract infections [1,2]. The estimated prevalence ranges from 1 in 10,000 to 1 in 20,000 live births, although the condition is likely underdiagnosed, particularly in resource-limited settings [1,3]. Approximately 50% of affected individuals exhibit situs inversus due to abnormal embryologic ciliary function, forming the basis of Kartagener syndrome [4,5]. This syndrome is classically defined by the triad of bronchiectasis, chronic sinusitis, and situs inversus [4]. However, incomplete or atypical forms lacking one component of the triad have been increasingly recognized and may contribute to delayed diagnosis, especially in adulthood [3,6]. Imaging, particularly computed tomography, plays a central role in identifying the characteristic pulmonary and anatomical features of this condition [7]. We present a case of atypical Kartagener syndrome diagnosed at the age of 40 years, with emphasis on radiologic findings (Fig. 1).

**Fig. 1:**
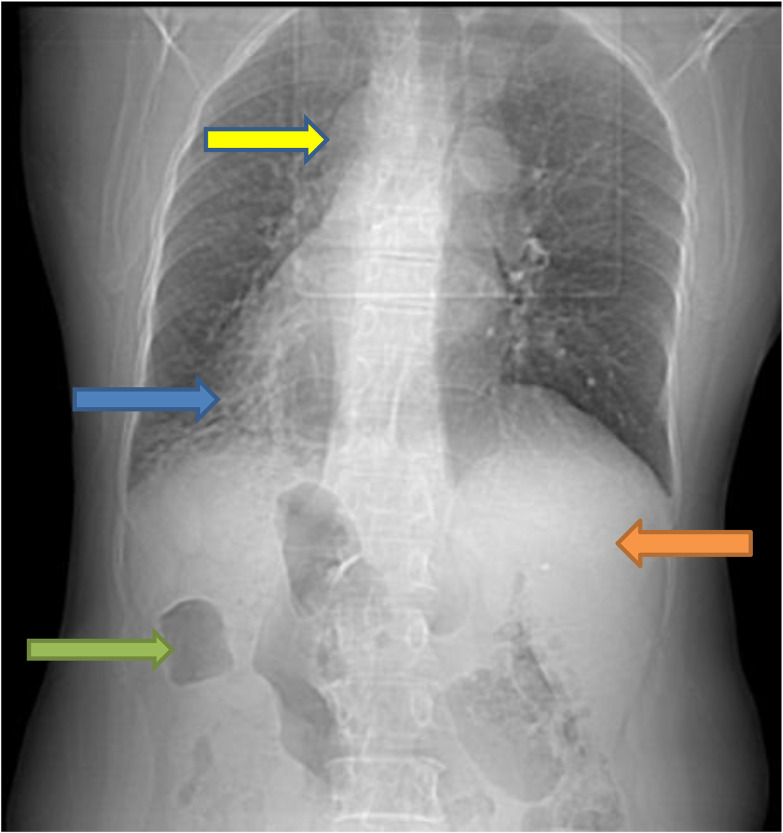
PA chest radiograph demonstrates the cardiac apex (blue arrow) pointing to the right with a right-sided aortic arch (yellow arrow). On the included upper abdomen, the liver shadow(orange arrow) is seen on the left and the gastric bubble (green arrow) on the right, consistent with situs inversus. There are bilateral mid- and lower-lung zone perihilar focal areas of mild bronchiectasis with increased reticular opacities.

## Case presentation

A 40-year-old female presented with productive cough, shortness of breath, pleuritic chest pain, and fever. She reported a long history of recurrent respiratory symptoms beginning at the age of 17 years, characterized by repeated episodes of productive cough requiring multiple courses of antibiotics, which resulted in temporary improvement followed by recurrence within two to three months. The patient also reported a history of primary infertility, with no prior successful pregnancies despite prolonged periods of unprotected intercourse. There was no history suggestive of chronic sinusitis or imaging findings to suggest sinusitis (Fig. 4).

The patient had not previously been diagnosed with Kartagener syndrome or primary ciliary dyskinesia. Prior management consisted of repeated courses of antibiotics and symptomatic treatment for recurrent lower respiratory tract infections at various healthcare facilities. Despite longstanding respiratory symptoms beginning in adolescence, no prior investigation for an underlying ciliary motility disorder had been performed. The current presentation at 40 years of age represented the first recognition of the constellation of findings suggestive of Kartagener syndrome.

Computed tomography of the chest revealed dextrocardia with complete situs inversus (Fig. 2A-C). There was bilateral bronchiectasis predominantly involving the middle lobe and lingula, associated with bronchial wall thickening and mucus impaction. In addition, multiple areas of tree-in-bud nodularity were noted, consistent with small airway inflammation, along with patchy ground-glass opacities in the adjacent lung parenchyma (Fig. 3A-D). The constellation of imaging findings, in conjunction with the clinical history of recurrent respiratory infections and infertility, was highly suggestive of an underlying ciliary motility disorder consistent with an atypical presentation of Kartagener syndrome.

**Fig. 2:**
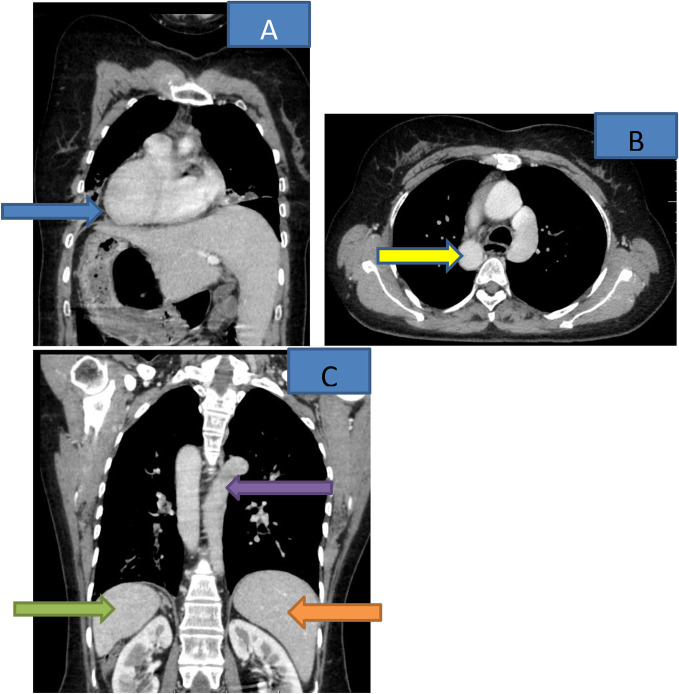
Coronal mediastinal window chest CT images (A–C) demonstrate the cardiac apex (blue arrow) on the right with a right-sided aortic arch (yellow arrow) (A, B) and right-sided descending aorta. A left-sided inferior vena cava with azygos continuation is also noted (purple) (C). The upper abdominal organs show situs inversus, with the liver (orange arrow) on the left and the spleen (green arrow) on the right (A, C).

**Fig. 3:**
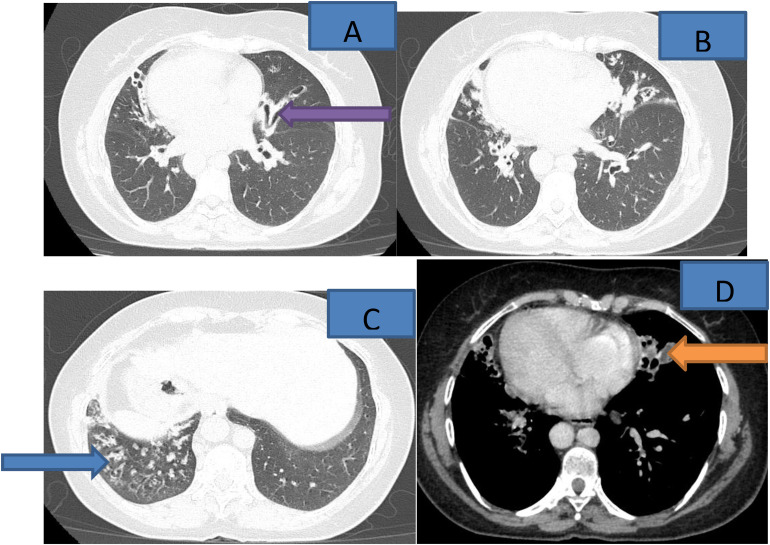
Axial chest CT lung window images at different levels (A–C) demonstrate mild bronchiectasis (purple arrow) involving the lingular segment and the middle-lobe equivalent in this patient with bilobed right and left lungs (mirror-image anatomy), with associated bronchial wall thickening and surrounding tree-in-bud opacities (blue arrow). Axial mediastinal window image (D) shows bronchiolar dilatation with mucous impaction (orange arrow).

**Fig. 4:**
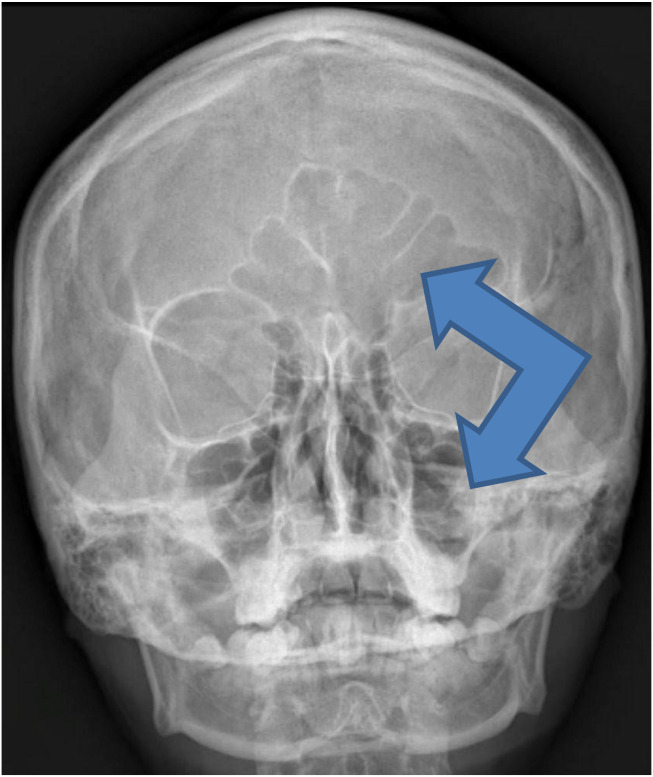
AP radiograph of the paranasal sinuses shows normal sinus outlines without opacification or mucoperiosteal thickening (blue arrow).

Following diagnosis, the patient was treated for the acute respiratory exacerbation with antibiotic therapy, bronchodilator treatment, and airway clearance measures. She was counseled regarding the chronic nature of the disease and referred for pulmonary follow-up for long-term management of bronchiectasis. Recommendations included regular airway clearance therapy, vaccination against respiratory pathogens, and prompt treatment of future respiratory infections. Further evaluation for primary ciliary dyskinesia using specialized diagnostic testing was recommended if resources became available. Counseling regarding the association between primary ciliary dyskinesia and infertility was also provided.

## Discussion

Primary ciliary dyskinesia is characterized by impaired motility of respiratory cilia, resulting in ineffective mucociliary clearance, chronic airway infection, and progressive structural lung damage [1,2]. Bronchiectasis is a hallmark feature and often demonstrates a characteristic distribution involving the middle lobe and lingula, as observed in this case [7]. Epidemiologically, situs inversus occurs in approximately 50% of patients with primary ciliary dyskinesia, reflecting the role of motile cilia in embryologic left-right axis determination [4,5]. Despite these recognizable features, diagnosis is frequently delayed, with many patients remaining undiagnosed until adulthood due to variability in clinical presentation and limited access to specialized diagnostic testing [3,6].

In the present case, the diagnosis was not suspected for more than two decades despite recurrent respiratory symptoms and infertility. The absence of chronic sinusitis may have contributed to the delayed recognition of the underlying disorder. This highlights the importance of considering atypical forms of Kartagener syndrome in adults presenting with recurrent respiratory infections and situs abnormalities, even when the complete classical triad is absent.

From a radiologic standpoint, computed tomography is essential for detecting bronchiectasis, evaluating its distribution, identifying mucus plugging, and demonstrating associated features such as tree-in-bud nodularity and ground-glass opacities [7]. Additionally, CT allows clear visualization of situs anomalies, which are key diagnostic clues. The presence of infertility in this patient further supports the diagnosis, as impaired ciliary motility in the fallopian tubes can result in defective ovum transport in females with primary ciliary dyskinesia [4,2].

Differential diagnoses such as cystic fibrosis and post-infectious bronchiectasis were considered; however, the combination of situs inversus, long-standing symptoms, infertility, and characteristic imaging findings favors a diagnosis within the spectrum of primary ciliary dyskinesia [6,8]. A limitation of this case is the lack of confirmatory diagnostic testing, including nasal nitric oxide measurement, high-speed video microscopy, transmission electron microscopy, or genetic analysis. Nevertheless, the clinical and radiologic findings are highly suggestive of Kartagener syndrome.

## Conclusion

This case demonstrates the characteristic computed tomography findings of an atypical presentation of Kartagener syndrome in an adult patient. Recognition of bronchiectasis with middle lobe and lingular predominance in the setting of situs inversus should prompt consideration of an underlying ciliary motility disorder. Radiologists play a crucial role in suggesting the diagnosis of primary ciliary dyskinesia, even in the absence of the full clinical triad, thereby helping to reduce diagnostic delay and guide appropriate management.

## Patient consent

Written informed consent were obtained from the patient for publication of this case report and the associated images.
